# Theoretical Insights into the Ultrafast Deactivation Mechanism and Photostability of a Natural Sunscreen System: Mycosporine Glycine

**DOI:** 10.1021/acs.jpca.3c02360

**Published:** 2023-05-30

**Authors:** Reza Omidyan, Leila Shahrokh, Abigail L. Whittock, Vasilios G. Stavros

**Affiliations:** † Department of Chemistry, University of Isfahan, Isfahan 81746-73441, Iran; ‡ Department of Chemistry, University of Warwick, Coventry CV4 7AL, U.K.; § Analytical Science Centre for Doctoral Training, Senate House, University of Warwick, Coventry CV4 7AL, U.K.; ∥ School of Chemistry, University of Birmingham, Edgbaston, Birmingham B15 2TT, U.K.

## Abstract

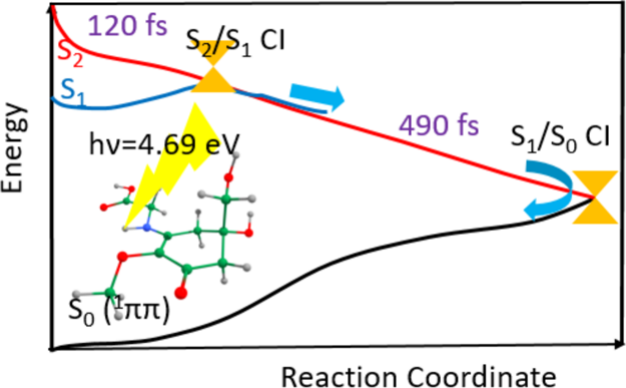

In this work, different levels of quantum computational models such as MP2, ADC(2), CASSCF/CASPT2, and DFT/TD-DFT have been employed to investigate the photophysics and photostability of a mycosporine system, mycosporine glycine (MyG). First of all, a molecular mechanics approach based on the Monte Carlo conformational search has been employed to investigate the possible geometry structures of MyG. Then, comprehensive studies on the electronic excited states and deactivation mechanism have been conducted on the most stable conformer. The first optically bright electronic transition responsible for the UV absorption of MyG has been assigned as the S_2_ (^1^ππ*) owing to the large oscillator strength (0.450). The first excited electronic state (S_1_) has been assigned as an optically dark (^1^nπ*) state. From the nonadiabatic dynamics simulation model, we propose that the initial population in the S_2_ (^1^ππ*) state transfers to the S_1_ state in under 100 fs, through an S_2_/S_1_ conical intersection (CI). The barrierless S_1_ potential energy curves then drive the excited system to the S_1_/S_0_ CI. This latter CI provides a significant route for ultrafast deactivation of the system to the ground state *via* internal conversion.

## 1 Introduction

Ultraviolet radiation (UVR) is the most energetic region of the broad spectrum of wavelengths from solar radiation that reaches the earth. This radiation is further subdivided into UVA (400–315 nm), UVB (315–280 nm), and UVC (280–100 nm).^1,2^ Almost all the UVC and a large fraction of the UVB radiation are absorbed by the ozone layer in the stratosphere. This results in 5% UVB and 95% UVA accounting for the total UVR that reaches the earth’s surface. These energy components have an impact on the earth’s biosphere.^3^ Although UVR can be beneficial, for example, the production of vitamin D being essential for prevention of skeletal disease,^4−6^ there are lots of concerns regarding its hazardous effects on life.^7−10^ The damaging effects of overexposure to UVR have been widely reported in previous studies and reviews, including cataract formation, skin aging, DNA mutation, and skin cancer.^11−15^ Hence, the need for a balance between exposure to UVR and protection against overexposure to UVA and UVB is of crucial importance.^16^

There are different types of photoprotective compounds present on the market. Nevertheless, so far, no compound has proved to be exceptionally versatile. A major concern regarding photoprotective systems (sunscreens) is that some of the active ingredients within sunscreens are inherently not photostable. A well-known example of this issue is the UV filter avobenzone.^17^ A good candidate for an efficient UV-filter (the active ingredient within sunscreens) should have efficient nonradiative decay and long-term photostability to dissipate the excess absorbed energy safely and quickly to regenerate the original ground-state molecule ready for reabsorption of another photon.^12^

Photoprotection in micro- and macroorganisms is achieved by a number of different molecules.^18^ The particular family of molecules of interest here are mycosporines and mycosporine-like amino acids (MAAs). Mycosporines are derived from a cyclohexenone core, and MAAs are derived from a cyclohexenimine core.^19^ Currently, there are over 70 mycosporines and MAAs isolated, and the various substituents on the ring, for example, the amino and imino functionalities, which are often amino acids or amino alcohols, are responsible for the wide range in peak absorption wavelength across both UVA and UVB regions of the electromagnetic spectrum.^20^ These properties ideally lend themselves toward use in future cosmeceutical applications, largely due to the ability to tune the range (UVA and UVB) over which particular mycosporines and MAAs absorb. In contrast to the crucial photoprotection role that they have, rare reports in the literature are devoted to the fundamental root of the photophysical nature of these systems especially involving the cyclohexenone core.^21−23^ Recent studies on MAAs clearly show that the excited electronic states are very short-lived, decaying rapidly to repopulate the ground electronic state. However understanding of the mechanisms of the relaxation dynamics in mycosporines and MAAs is still in its infancy.^24,25^

**Figure 1:**
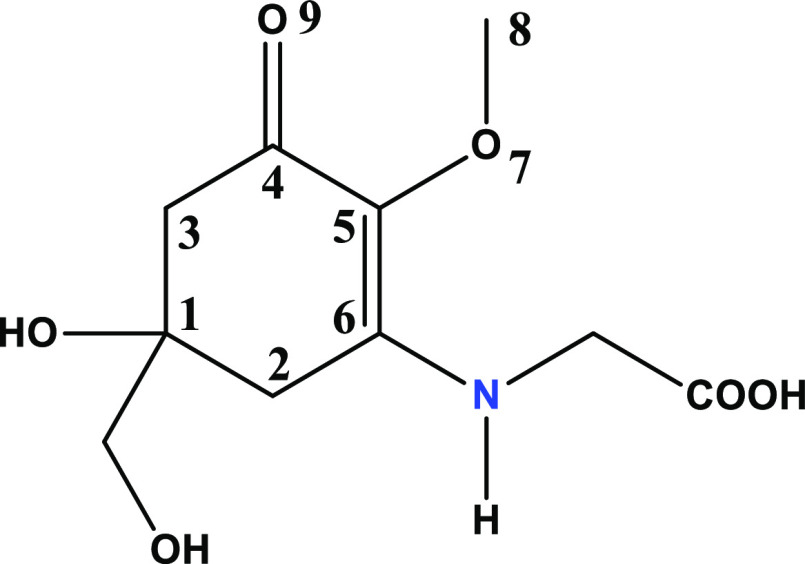
Chemical structure and numbering pattern of MyG applied in this work. Hydrogen atoms over the ring and other C atoms have been omitted for clarity.

Herein, we report on a systematic investigation of the photochemical mechanisms for radiationless excited-state deactivation of the most stable conformer of mycosporine-glycine (abbreviated as MyG henceforth). This system has an absorption maximum at 310 nm in water solvent medium^26^ and possesses a cyclohexenone ring linked with an amino acid, glycine (Figure 1). However, to the best of our knowledge, there are no reports on the spectroscopy of this system in the gas phase. The motivation behind such studies is that it allows us to distinguish between intrinsic molecular properties and properties resulting from the biological environment.^23,27^ The details of fundamental biochemical characteristics and interactions are often hidden by macroscopic solvent effects and interactions with other molecules. Thus, studying individual biomolecules in vacuum reveals the intrinsic properties of the most basic biomolecular processes.^27,28^

In the present study, we explored the relevant excited-state potential-energy functions along a photochemical reaction path and optimized the relevant conical intersections (CIs) involved in the deactivation process. We have performed these explorations with the ADC(2) and CASSCF electronic-structure methods. In addition to the static exploration of the excited-state potential-energy profiles, we report the results of nonadiabatic trajectory-surface-hopping molecular dynamics simulations of MyG. Taking the implicit ethanol solvent model into account, the dynamics simulations were performed with the TD-DFT electronic-structure method, which is the method of choice for systems of this size.

## 2 Computational Details

The conformational landscapes of MyG have been determined based on the Monte Carlo multiple minimum (MCMM) methodology^29^ using the MacroModel suite of program (version 8.5).^29^ This initial exploration of the PES was done using classical molecular mechanics force fields (MMFFs).^30^ The optimized geometry of selected conformers of the ground state and corresponding vibrational frequencies have been determined either at the density functional theory (DFT)/B3LYP or RI-MP2 level of theory using the cc-pVDZ basis set.^31^ The vertical and adiabatic excitation energies of the lowest excited singlet states and oscillator strengths for electronic transitions were computed based on the RI-ADC(2), TD-DFT/ωB97XD,^32^ and multistate complete active space second-order perturbation (MS-CASPT2)^33^ theory. The RI-MP2 and RI-ADC(2) calculations were carried out by the Turbomole program suit (V 6.3.1).^34,35^ The DFT and TD-DFT calculations were employed using the Gaussian 16 program,^36^ and CASSCF/CASPT2^37,38^ computations were performed using OpenMolcas (V 18.09).^39,40^

We have determined the potential energy curves representing the deactivation mechanism of our selected system based on linear interpolation in internal coordinates (LIIC). Our method of choice in this case is ADC(2) because of its reliability from our previous works along with works from other groups.^41−46^ In addition, CI geometries were optimized using a state-averaged complete active space self-consistent field (SA-CASSCF).^47^

Moreover, determination of the UV absorption spectrum and nonadiabatic dynamics simulation have been conducted based on the TD-DFT model using Newton-X (V. 2.0)^48^ interfaced with Gaussian 16. The validity of the selected model for determining the excited-state deactivation processes has been confirmed in different reports.^16,49−52^ To perform nonadiabatic dynamics simulations, the initial conditions were computed for geometry and nuclear momenta sampled from a Wigner distribution based on the S_0_ normal modes. Vertical excitation energies were computed at the ωB97XD/6-31G* level, and these energies were obtained with δ = 0.05 eV Lorentzian line broadening for the two lowest singlet excited states.

In addition, to investigate the solvent’s influence on absorption spectra, the polarizable continuum model (PCM/ethanol model)^53^ implemented in Gaussian 16 has been used.

## 3 Results and Discussion

### 3.1 Ground-State Optimized Geometries

The conformational flexibility of MyG mainly arises from (1) the −glycine side chain, (2) the −methoxy group (−OCH_3_), (3) the hydroxymethyl group (−CH_2_OH), and (4) flexible carbon sites of the ring. The orientation in space of the glycine side chain with respect to the six-membered ring could be subject to diversity in conformation in addition to location of its own atoms with respect to each other. Over the cyclohexenone ring, there are two double bonds, C_5_=C_6_ and C_4_=O_9_; thus, the C_4_, C_5_, and C_6_ locates mainly in the same plane owing to the sp^2^ hybridization nature of the C atoms (see Figure 1 for numbering). Other carbon atoms in the ring (C_1_, C_2_, and C_3_) are sp^3^-hybridized, and there are two possibilities for out-of-plane location of the C_1_ atom and its subgroups; it could be pointing upward or pointing downward of the ring. Consequently, this offers another root of obtaining new conformations.

**Figure 2:**
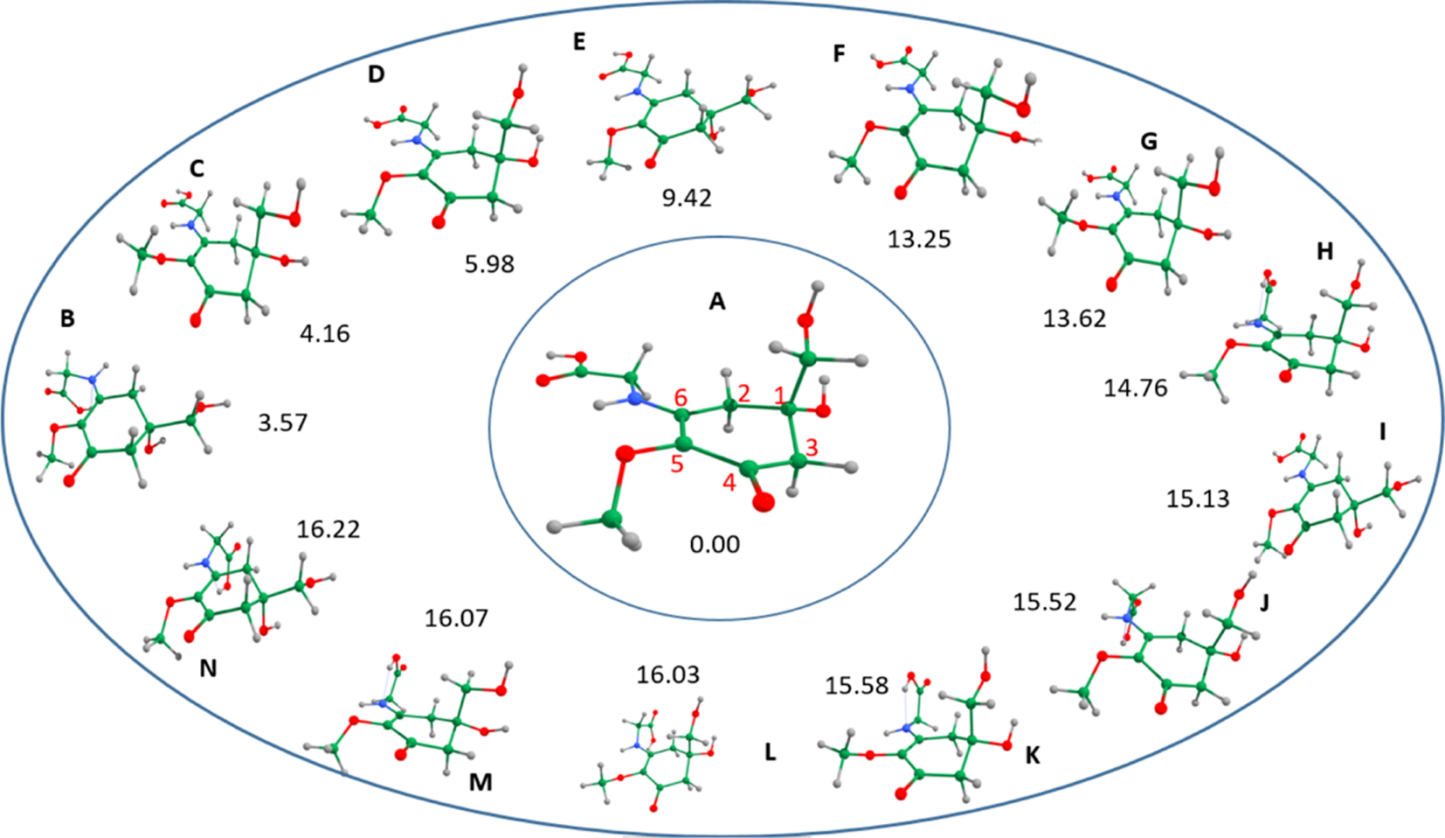
Optimized structures of the 14 most stable structures of MyG at the B3LYP/cc-pVDZ level of theory (the energetic values have been corrected by ZPE). The most stable structure (MyG-A) is shown in the center with other structures labeled according to decreasing stability. Their energies (in kJ mol^–1^) are relative to MyG-A.

The conformational landscape of MyG has been determined based on the conformation search algorithm using the MCMM methodology. MMFFs were used for 1000 steps. All structures generated with energies within 20.0 kJ mol^–1^ of the minimum were examined. Based on the MCMM theoretical model, 101 conformers have been obtained. We have selected 30 of the most stable structures and determined the minimum geometry and consequently the relative stabilities based on the DFT method (at the B3LYP/cc-pVDZ level). Optimized geometries of the selected lowest-lying conformers (in energy) are presented in Figure 2. As shown, we have called the most stable structure MyG-A, which is 3.57 kJ mol^–1^ more stable than the next nearest conformer (at the B3LYP/cc-pVDZ level of theory, taking the ZPE correction into account). Comprehensive information regarding the 30 conformers is also presented in Table S1, Supporting Information file.

As shown in Figure 2, MyG-A exhibits a puckering feature around C_1_. The dihedral angle of C_1_–C_2_–C_3_–C_4_ describing this deformation from the ring has been predicted at 49°. Other carbon sites of the six-membered ring stay roughly on the same plane; thus, the system looks like a half-chair. In addition, the dihedral angle of C_15_–O_10_–C_5_–C_6_ describing the position of the −O–CH_3_ side chain with respect to the cyclohexenone ring has been predicted at 115° which is near-perpendicular (downward). The glycine side chain remains in the same plane with respect to the six-membered cyclohexenone ring. Moreover, from inspection of Figure 2, it is apparent that the out-of-plane deformation of C_1_ above the molecular plane is present in several structures (for instance, A, C, D *etc.*), while its out-of-plane deformation below the molecular plane is also significantly populated (such as B, E, I, L, and N). Furthermore, when the glycine side chain remains in the molecular plane, the corresponding conformers are significantly more stable than those that have other orientations (such as K, L, M, and N).

To note, we have optimized the 6 most stable conformers based on the MP2/cc-pVDZ theoretical model, with their relative stabilities confirming the DFT level results. The optimized structure of the most stable MyG-A conformer at the MP2 level has been determined to be fairly similar to that found with DFT, with slight differences in the angles and dihedrals, especially in the glycine side chain. Comparison of the optimized MP2 and DFT/B3LYP structure of MyG-A is shown in Figure S1 of the Supporting Information.

From our conformational study, we have selected the most stable conformer (MyG-A) and concentrated on its electronic structure, excited states, and deactivation mechanism which now follows.

### 3.2 Electronic Transition Energies

Calculated vertical transition energies and oscillator strengths of the most stable conformer, MyG-A, to the four lowest lying singlet excited states (S_1_–S_4_) have been determined and are presented in Table 1. We have employed different levels of theory: TD-DFT using the ωB97XD functional; RI-ADC(2); and MS-CASPT2. Regarding the TD-DFT calculations, the ωB97XD functional was used as it successfully described the energy of the electronic states and nonradiative deactivation process in similar systems.^24,49,51,54−56^

**Table 1:** Vertical Transition Energies and Oscillator Strengths for the Most Stable Conformer MyG-A in the Gas Phase, Determined at the TD-ωB97XD, ADC(2), and MS-CASPT2 Levels of Theory with cc-pVDZ Basis Sets^a^

transition energy/eV (MyG-A)	TD-ωB97XD		ADC(2)		MS-CASPT2	
excited state	Δ*E* (f)	character	Δ*E* (f)	character	Δ*E* (f)	character
S_1_	4.157 (0.0059)	nπ_1_^*^	4.031 (0.0004) ***2.86***^b^	nπ_1_^*^	4.050 (0.000)	nπ_1_^*^
S_2_	4.709 (0.3922)	ππ_1_^*^	4.693 (0.4497)	ππ_1_^*^	4.680 (0.7600)	ππ_1_^*^
S_3_	5.512 (0.0131)	ππ_2_^*^	6.182 (0.0066)	ππ_2_^*^	7.150 (0.011)	ππ_2_^*^
S_4_	6.097 (0.0005)	nπ_2_^*^	6.264 (0.0005)	nπ_2_^*^	7.540 (0.003)	nπ_2_^*^ + nσ*

a The MS-CASPT2 results have been obtained based on 8 electrons and 8 orbitals as the active space and 5 multistates.

b Adiabatic S_1_ ← S_0_ transition energy determined at the optimized S_1_ geometry of MyG-A. Further information regarding the MOs and transition characters could be found in Table S3, Supporting Information file.

**Figure 3:**
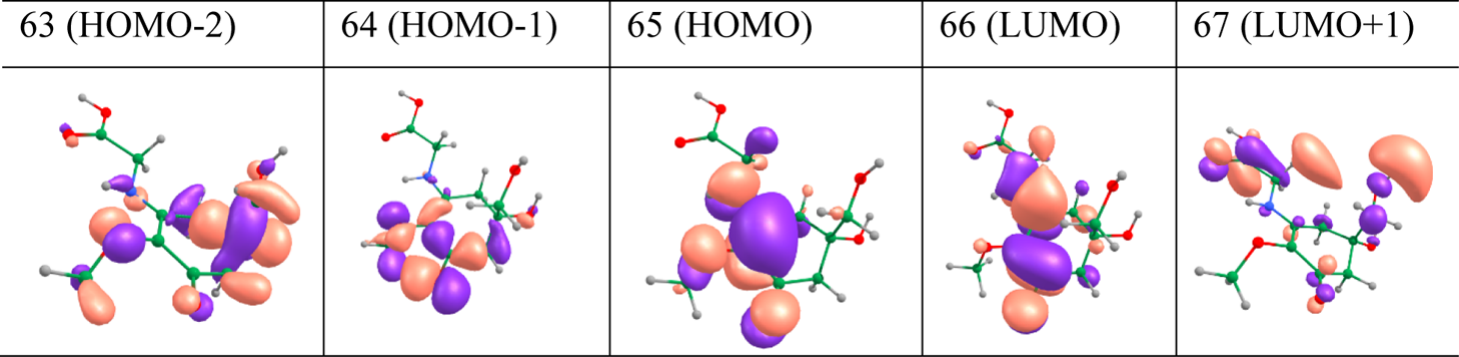
Selected valance MOs of MyG-A, determined at the SCF/cc-pVDZ level of theory, playing a prominent role in the lowest lying electronic transitions.

Selected valence molecular orbitals (MOs) of MyG-A are presented in Figure 3. According to our ADC(2) results, the S_1_ ← S_0_ electronic transition has been assigned as an optically dark (^1^nπ*) transition, originating from LUMO ← HOMO – 1 (85%) single-electron transitions. The HOMO and LUMO, respectively, stand for the highest occupied molecular orbital and the lowest unoccupied molecular orbital. The second singlet electronic transition (the S_2_ ← S_0_) has been assigned as an optically bright ^1^ππ* transition, involving a LUMO ← HOMO transition (92%). As the molecular point group is C1 (and there is no symmetry plane), our assignments are only qualitative.

The MOs involving the S_3_ ← S_0_ electronic transition have been assigned mainly as LUMO + 1 ← HOMO and LUMO + 2 ← HOMO transitions, and for S_4_ ← S_0_, the LUMO ← HOMO – 3 transition has been predicted to be the main contributor. More information regarding the electronic transitions and contributing MOs can be found in the Supporting Information.

The MS-CASPT2 results have been obtained, selecting the 8 electrons and 8 valance orbitals (4 occupied and 4 virtual) as the active space (see Table S2, Supporting Information). We have examined different active spaces to determine the vertical transition energies. As the S_2_–S_0_, ^1^ππ* electronic transition dominates the photophysics of MyG, we have selected the active space yielding the most reliable result for this electronic transition. Although there is no experimental UV absorption spectrum of MyG in the gas phase, as mentioned before, the λ_max_ of absorption around 300 nm has been reported for MyG in water solution. The active space of (8, 8) gives better results than others, and no improvement has occurred considering the larger active spaces (see Table S4, Supporting Information file).

As shown in Table 1, the MS-CASPT2 results for S_1_ and S_2_ states are comparable with the ADC(2) and also TD-DFT/ωB97XD results. There is a discrepancy relating to the S_3_ ← S_0_ electronic transition energy which is significantly underestimated at the TD-DFT level. However, due to the optically dark nature of this state (low oscillator strength), it should have no significant bearing on the photophysics of MyG. The energetic value of the S_2_ ← S_0_ transition (^1^ππ*), responsible for the UV absorption of MyG-A, has been determined as 4.71, 4.69, and 4.68 eV, respectively, at the TD-ωB97XD, RI-ADC(2), and MS-CASPT2 levels of theory. Good agreement of TD-ωB97XD results with RI-ADC(2) and MS-CASPT2 (especially for the S_1_ and S_2_ transition energies) indicates that this functional is sufficiently reliable to describe electronic structures of the selected systems.

To perform a comparison between the five most stable structures of MyG-(A, B, C, D, and E), we have determined the vertical electronic transition energies in the gas phase and also with the implicit ethanol solvent for the four lowest lying singlet excited states at the TD-ωB97XD/cc-pVDZ level of theory. The results are presented in Table 2. Inspection of these results reflects at least two important points:

(1) The electronic transition energies of the five most stable conformers of MyG are quite close to each other. This indicates that the photophysics of these systems should be similar.
(2) The electronic transition energies in the implicit solvent (ethanol) are mildly different from the corresponding gas-phase results. Consistent with previous results,^57^ it indicates that the solvent effect on the photophysical nature of these systems would be negligible.

**Table 2:** Vertical transition energies and oscillator strengths for lowest lying electronic transitions in the five most stable conformers of MyG^a^

transition energy/eV (TD-ωB97XD/cc-pVDZ)					
excited state	MyG-A	MyG-B	MyG-C	MyG-D	MyG-E
Gas Phase					
S_1_ (nπ_1_^*^)	4.157 (0.0059)	4.091 (0.0062)	4.083 (0.0051)	4.018 (0.0057)	4.165 (0.0098)
S_2_ (ππ_1_^*^)	4.709 (0.3922)	4.709 (0.391)	4.749 (0.3956)	4.593 (0.3828)	4.7421 (0.3974)
S_3_ (ππ_2_^*^)	5.512 (0.0131)	5.460 (0.0125)	5.525 (0.0102)	5.453 (0.0129)	5.419 (0.0161)
S_4_ (nπ_2_^*^)	6.097 (0.0005)	6.091 (0.0005)	6.095 (0.0005)	5.692 (0.0016)	6.0921 (0.0005)
PCM/Ethanol					
S_1_ (nπ_1_^*^)	4.336 (0.0327)	4.279 (0.0298)	4.266 (0.0245)	4.206 (0.0320)	4.328 (0.0414)
S_2_ (ππ_1_^*^)	4.603 (0.4513)	4.594 (0.4543)	4.630 (0.4613)	4.485 (0.4403)	4.662 (0.4608)
S_3_ (ππ_2_^*^)	5.898 (0.0112)	5.873 (0.0103)	5.921 (0.0090)	5.704 (0.0066)	5.867 (0.0110)
S_4_ (nπ_2_^*^)	6.191 (0.0006)	6.189 (0.0006)	6.193 (0.0006)	5.862 (0.0007)	6.443 (0.0397)

a Calculations were performed in the gas phase and in the PCM/ethanol implicit solvent, determined at the TD-ωB97XD/cc-pVDZ level of theory.

**Figure 4:**
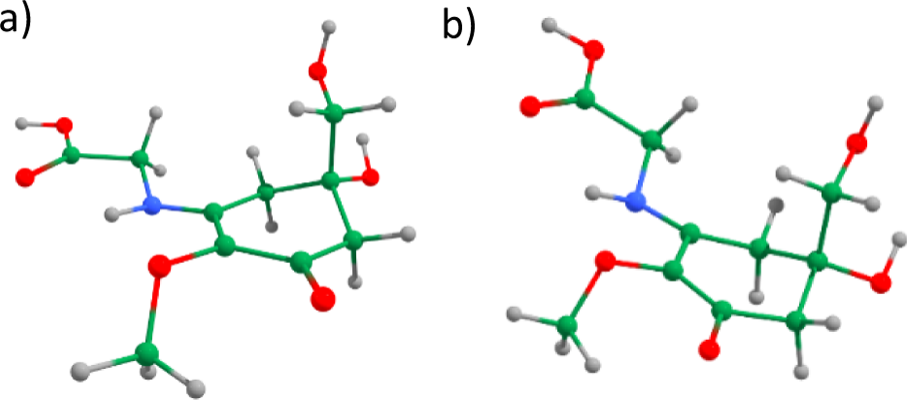
Optimized geometries of (a) S_0_ ground state and (b) S_1_ (^1^nπ*) state determined, respectively, at the MP2 and ADC(2) theoretical levels.

To garner more insights into the exited-state nature of MyG-A, we have performed the RI-ADC(2) geometry optimizations for the excited S_1_ and S_2_ states. As mentioned *supra*, the S_1_ is an optically dark state having ^1^nπ* character and roughly zero oscillator strength. The S_2_ (^1^ππ*) state is responsible for the UV absorption of MyG (*F* = 0.450 in MyG-A). The S_1_ excited state exhibits a local minimum, with a molecular geometry that is significantly different from that of the ground state (see Figure 4a,b). The glycine side chain exhibits an out-of-plane movement, and there is a slight distortion of the ring around the C_6_ and C_3_ regions. These changes in geometry stabilize the S_1_ excited state, so that the adiabatic S_1_ ← S_0_ transition energy has been predicted to be significantly lower (2.86 eV) than its vertical analogue (4.03 eV). Furthermore, our calculations predict no local minimum on the S_2_ potential energy surface, and the S_2_ geometry optimization results in strong deformation of the six-membered ring from the C_6_ carbon site and other side chains.

### 3.3 Photophysical Behaviors: CIs and Potential Energy Profiles

**Figure 5:**
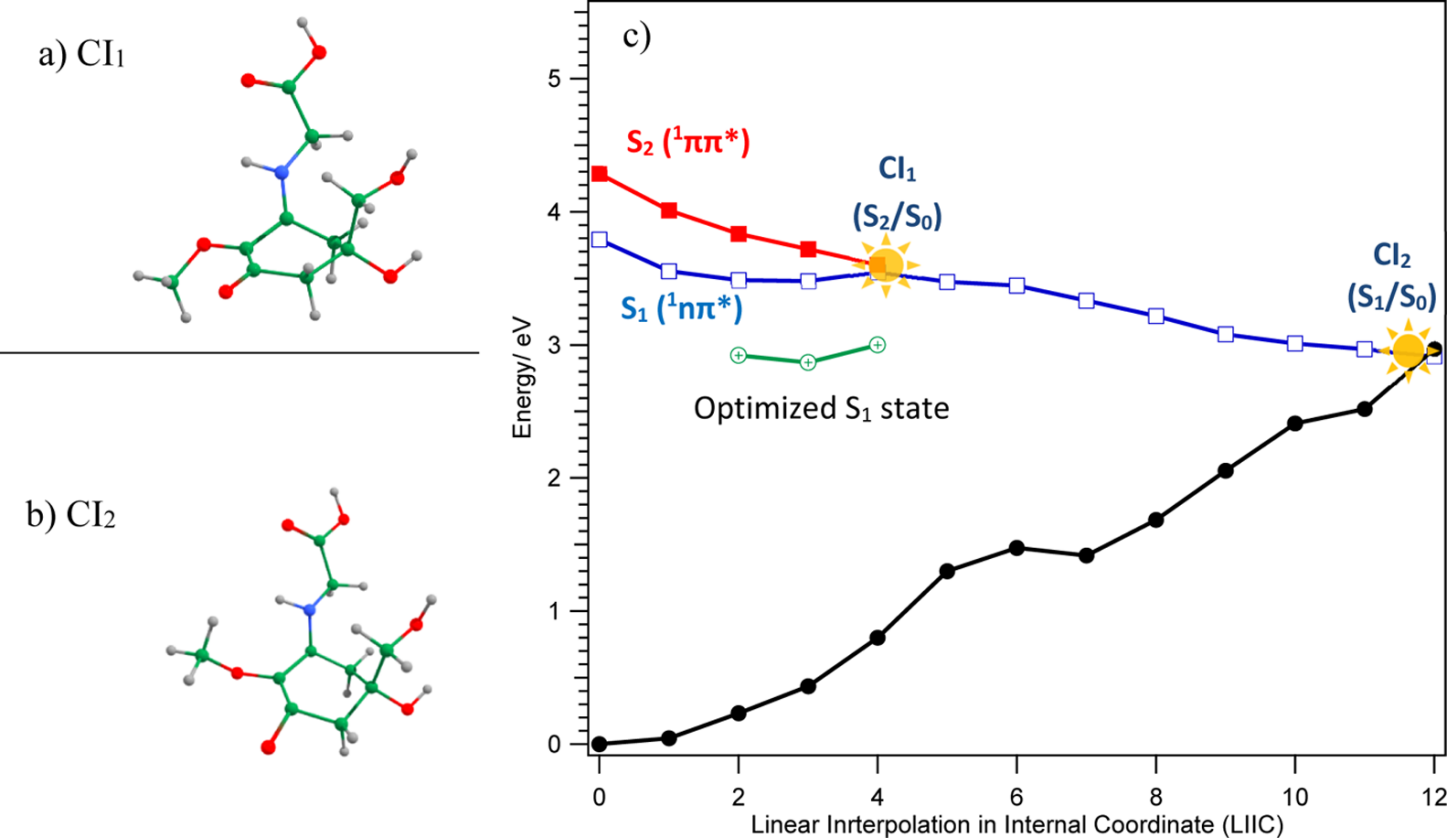
Optimized geometries of the CIs located for MyG-A: (a) CI_1_ (S_2_/S_1_) and (b) CI_2_ (S_1_/S_0_) determined at the SA-CASSCF(6,6)/cc-pVDZ theoretical level. (c) PE profile of the ground (black) and 2 singlet excited states of MyG-A calculated at the ADC(2)/cc-pVDZ level of theory along the LIIC reaction path.

To gain more insights into the excited-state relaxation mechanism of MyG-A, we have searched for possible CIs. We have located two CIs for MyG-A based on the SA-CASSCF(6,6)/cc-pVDZ theoretical model (see Figure 5a,b). The active space in the SA-CASSCF calculations contains 6 electrons in 6 MOs (3 occupied and 3 virtual MOs, Table S2, Supporting Information). The CI_1_ represents the S_2_/S_1_ potential energy crossing, and the CI_2_ describes the minimum structure of the S_1_/S_0_ curve crossing. As shown in Figure 5, both optimized CI structures exhibit puckering of the ring at C_6_, in addition to out-of-plane movement of the glycine moiety. This deformation is more pronounced in CI_2_. Moreover, there is a significant alteration in the C_8_–O_7_–C_5_–C_6_ dihedral angle (from 115.6° in the ground state to −113.7°). On the other hand, in CI_2_, the methoxy (−OCH_3_) group moves toward the glycine side chain (upward the ring). This alteration is accompanied by a slight out-of-plane distortion of C_5_ downward the ring. The optimized Cartesian coordinates of the CIs are presented in the Supporting Information.

It is well known that locating CIs does not warrant the nonradiative deactivation process since the presence of a large barrier could prevent this deactivation pathway. Thus, we have determined potential energy (PE) profiles for the ground and two electronic excited states (S_1_ and S_2_) based on the LIIC connecting the Franck–Condon (FC) region of MyG-A to CI_2_. The results are presented in Figure 5. As shown, the S_1_ and S_2_ potential energy profiles decrease in energy along the LIIC coordinate. The S_2_ potential energy curve first crosses with the S_1_ potential energy curve (obtaining the CI_1_ in the multidimensional picture).

This trend continues until the end of the reaction coordinate. The ground-state PE profile increases in energy along the LIIC coordinate, ultimately crossing the S_1_ PE profile. As shown, the S_1_ PE curve starting from the FC region passes through a shallow local minimum and then increases to cross with the S_2_ PE curve. This local minimum in the LIIC potential energy profile is in accordance with the results of obtaining a minimum geometry for the S_1_ state as discussed *supra*. The S_1_/S_0_ curve crossing at the end of the reaction coordinate provides an essential root for ultrafast deactivation of the excited state based on internal conversion to the ground-state S_0_ potential energy surface. Since the predicted CI (around 3.0 eV) is located significantly lower than the S_2_ vertical transition energy [of 4.69 eV at the ADC(2) level], one could envisage that excited-state population in S_2_ will approach the CI especially since the S_2_ PE profile is purely repulsive.

One may be concerned that ADC(2), as a single-reference method, would not be reliable enough to describe the photophysical nature, especially the S_1_/S_0_ CI of MyG-A. To address this concern, we have determined the ground- and excited-state potential energy profiles of MyG-A along the LIIC coordinate (corresponding to Figure 5), based on the MS-CASPT2/CASSCF(6,6)/cc-pVDZ theoretical model. The results have been presented in Figure S2, Supporting Information file. As shown, the CASPT2 results support the validity of our ADC(2) theoretical results. To add, previous reports have also demonstrated that ADC(2) results are qualitatively reliable in describing the photophysics of organic systems.^41,42,44,46,58−60^

### 3.4 Nonadiabatic Dynamics Simulations Results

In order to shed more light into the relaxation dynamics of MyG, we have employed the nonadiabatic dynamics simulation based on the TD-DFT theoretical model on the most stable conformer, MyG-A. In this regard, a nonadiabatic surface hopping dynamics simulation was performed from the second excited-state S_2_ (^1^ππ*) at the TD-ωB97xD/6-31G* level of theory, with ethanol as the implicit solvent.

**Figure 6:**
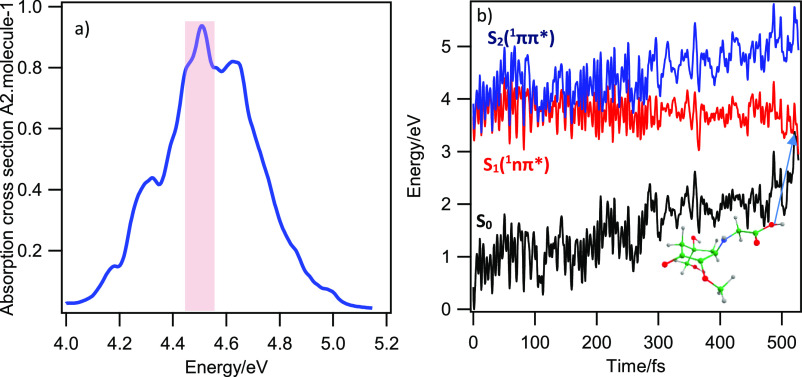
(a) UV absorption spectrum simulated at the TD-ωB97XD/6-31G* method based on the S_2_ ← S_0_ electronic transition in the PCM/ethanol implicit solvent with 300 points for MyG-A. (b) Energy profiles of a selected trajectory for MyG-A. The black, red, and blue curves, respectively, indicate the ground (S_0_), S_1_, and S_2_ excited states. The inset in panel a represents the geometry of S_1_/S_0_ CI (out-of-plane movement of glycine as the prominent alteration).

The photoabsorption spectrum of MyG-A, shown in Figure 6, was simulated using the nuclear ensemble approach.^61^ The velocity Verlet^62^ algorithm with a time step of 0.5 fs was used for the integration of Newton’s equations. Since the first bright state in MyG-A is the S_2_ (^1^ππ*) state, the nonadiabatic dynamics simulation has been started from S_2_, using the decoherence-corrected^63^ fewest switches surface hopping (DC-FSSH) approach.^64^ A total of 52 trajectories starting from the optically bright S_2_ state were considered. The initial conditions for the dynamics were sampled within the 4.55 ± 0.10 eV spectral window highlighted in Figure 6. We considered that a trajectory went back to the ground-state S_0_ when the S_1_–S_0_ energetic gap approached a threshold of 0.25 eV.

Surface hopping dynamics was simulated for a maximum of 1000 fs with a nuclear time step of 0.50 fs. 100% of optically bright S_2_ (^1^ππ*)-state population is transferred to the S_1_ (^1^nπ*) state within the initial ∼120 fs of the dynamics. Therefore, the deactivation mechanism starts at the S_2_ (^1^ππ*) state, with excited-state population traversing the S_2_/S_1_ CI in around 120 fs, leading the excited system to drive over the S_1_ PE curve ending by CI_2_ (see Figure 6b). This later CI provides an essential root for ultrafast internal conversion of the excited system to the ground state. Consequently, the net effect of this process would be converting the hazardous UV light (of 4.69 eV) to harmless vibrational motion (or heat). The dynamics results are in good agreement with our previously discussed *ab initio* results.

From the results of the dynamics, we predict that 82% of the trajectories relax based on the ring puckering from the C_6_ region (corresponding to CI_2_ presented in Figure 5b) as well as the C_3_ region. Moreover, 10% trajectories have been assigned to relax with ring puckering from C_1_ and C_4_ carbon sites.

To conclude this section, we present the time evolution of the nonadiabatic population of the ground and the first two excited states from the nonadiabatic dynamics simulation of MyG-A (Figures S3 and S4, Supporting Information). Using a sigmoid curve fitting procedure^56^ as an approximate guide, we determine that the S_2_ excited-state lifetime; τ_S_2__ is predicted to be ∼67 fs, while the S_1_ excited-state lifetime, τ_S_1__, is predicted to be ∼517 fs. Further details regarding this fitting is presented in Supporting Information, Figure S2. From this, we conclude that photoexcitation of MyG to S_2_ can relax to the S_0_ ground state in approximately τ_S_2__ + τ_S_1__ = 584 fs. This ultra-short lifetime of the excited state is in accordance with the high photostability of MyG-A.

## 4 Summary and Conclusions

Molecular mechanics configurational search has been employed to determine the possible structures and conformers of mycosporine glycine. We have employed stepwise computational models to determine the lowest lying conformers and consequently the most stable structure. Following this, state-of-the-art *ab initio* computational models as well as nonadiabatic dynamics simulations have been conducted to shed light on the photophysics of MyG. We show that the S_2_ (^1^ππ*) state is responsible for the UV absorption. Following photoabsorption in the UV region [4.69 eV at the ADC(2) theoretical level], excited population proceeds along a ring twisting reaction coordinate, traversing through the S_2_/S_1_ CI. This takes place on the order of 100 fs. We have also shown that the potential energy profile of the S_1_ state after the S_2_/S_1_ CI significantly decreases in energy and thus drives the excited system to the S_1_/S_0_ CI. This CI governs the ultrafast deactivation of the excited system to the ground state by internal conversion. These results provide significant molecular level insights into the origins of photostability of MyG and consequently its ability to photoprotect microorganisms against the potentially detrimental effects of UV radiation exposure. These results also provide benchmark data that could be used to develop next-generation UV filters for commercial applications.

## Supporting Information Available

Optimized geometry (along with relative energy in kJ mol^–1^) for 30 selected conformers of MyG, valance MOs contributing to the SA-CASSCF calculations of this work, *xyz* coordinates, lowest-lying transition energies, relevant MOs, and additional information about potential energy surfaces and time evolution of the nonadiabatic population of the ground state and first excited state from nonadiabatic dynamics simulation for MyG-A (PDF)
