# Supplementary material for: Theoretical Insights into the Ultrafast Deactivation Mechanism and Photostability of a Natural Sunscreen System: Mycosporine Glycine
Source: J Phys Chem A. 2023 May 30;127(22):4880–7. doi: 10.1021/acs.jpca.3c02360 (PMC10258844; doi:10.1021/acs.jpca.3c02360)
Supplement: Supplementary file 1 — jp3c02360_si_001.pdf [file jp3c02360_si_001.pdf]

Electronic Supplementary File

Theoretical Insights on the Ultrafast Deactivation Mechanism and Photostability of a Natural Sunscreen System: Mycosporine Glycine

Reza Omidyan[1,*], Leila Shahrokh[1], Abigail L. Whittock[2,3], and Vasilios G. Stavros[2,4]*

Department of Chemistry, University of Isfahan, 81746-73441, Isfahan, Iran
[2]*Department of Chemistry, University of Warwick, Coventry CV4 7AL, United Kingdom*
[3]*Analytical Science Centre for Doctoral Training, Senate House, University of Warwick, Coventry, CV4 7AL, United Kingdom*
[4]*School of Chemistry, University of Birmingham, Edgbaston, B15 2TT, United Kingdom*

This supplementary file contains Five tables (S1-S5) and Four Figures (S1-S4).

*Corresponding authors: r.omidyan@sci.ui.ac.ir, v.stavros@warwick.ac.uk

Table S1. *Optimized geometry (along with relative energy in kJ mol^{-1}) for 30 selected conformers of MyG determined at the DFT/B3LYP/cc-pVDZ level. The structures are labeled (on the top left corner) according to decreasing stability (the relative energy in kJ mol^{-1}, is presented at low right corner). Numbering of 1-14 most stable structures corresponds to A-N.*

1 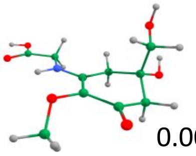 0.00	2 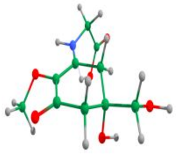 3.57	3 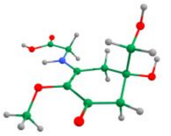 4.16	4 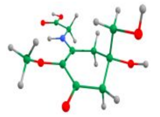 5.98
5 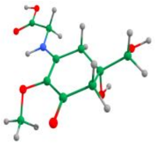 9.42	6 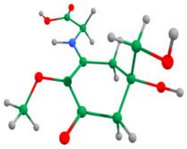 13.25	7 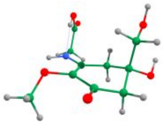 13.62	8 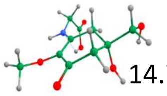 14.75
9 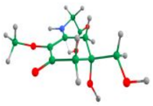 15.13	10 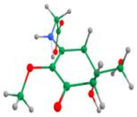 15.52	11 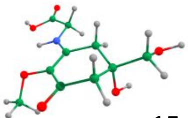 15.58	12 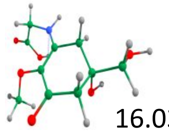 16.03
13 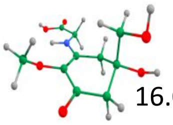 16.07	14 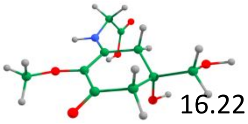 16.22	15 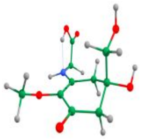 17.43	16 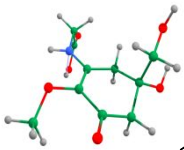 18.07
17 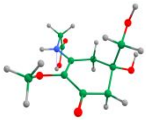 18.22	18 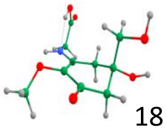 18.29	19 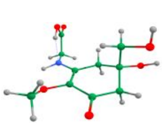 18.73	20 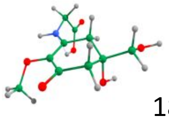 18.81
21 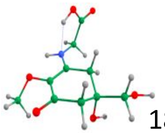 18.94	22 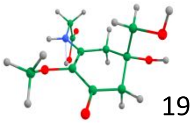 19.53	23 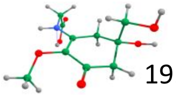 19.54	24 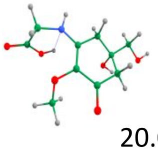 20.0

25 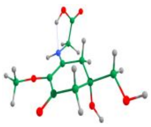 20.17	26 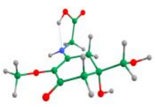 21.31	27 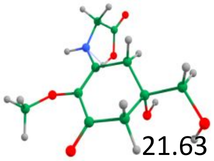 21.63	28 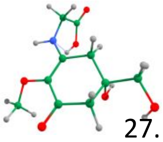 27.19
29 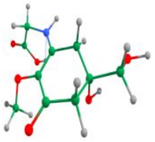 28.30	30 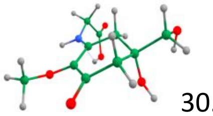 30.0		

Table S2: *Valance molecular orbitals contributing to the SA-CASSCF calculations of this work.*

66 (LUMO)	67	68	69
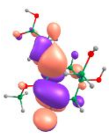	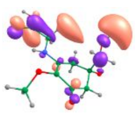	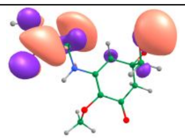	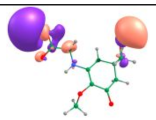
62	63	64	65 (HOMO)
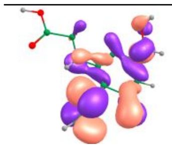	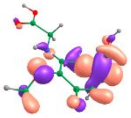	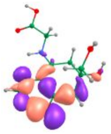	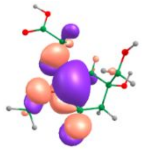

Table S3: *Contributed valance molecular orbitals in the S1-S4 electronic transitions determined based on different theoretical level.*

ADC(2)						
State	Contributions	HOMO-6	HOMO-1	HOMO	LUMO	LUMO+2
S_1	HOMO-1→LUMO	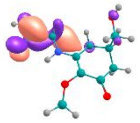	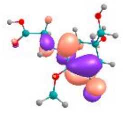	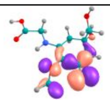	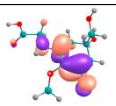	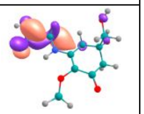
S_2	HOMO→LUMO					
S_3	HOMO→ LUMO+2					
S_4	HOMO-5→LUMO+2					
TD-DFT						
		HOMO-5	HOMO-1	HOMO	LUMO	LUMO+1
S_1	HOMO-1→LUMO	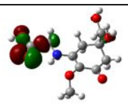	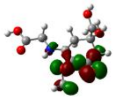	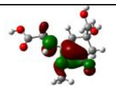	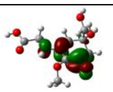	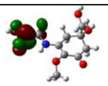
S_2	HOMO→LUMO					
S_3	HOMO→ LUMO+1					
S_4	HOMO-6→LUMO+2					
MS-CASPT2						
		HOMO-1	HOMO	LUMO	LUMO+1	LUMO+2
S_1	HOMO-1→LUMO	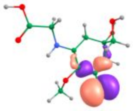	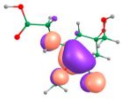	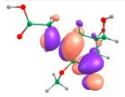	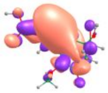	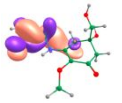
S_2	HOMO→LUMO					
S_3	HOMO→ LUMO+1					
S_4	HOMO→LUMO+2					

Table S4. *Active space influence on the lowest lying electronic transition of MyG-A. The energetic values are reported in eV.*

State\CASSCF Active space	(6,6)	(8, 8)	(10, 10)	(12, 12)
S_1	4.10	4.16	4.05	4.03
S_2	4.82	4.68	4.72	4.71
S_3	7.09	7.15	7.12	7.11
S_4	7.65	7.54	7.50	7.43

Table S5: *The xyz coordinates of the ground state optimized geometry of the five most stable conformations of MyG at the DFT/B3LYP/cc-pVDZ level of theory.*

MyG-A (MyG-A)

C	-2.02600	-1.13900	-0.34400
C	-0.52300	-1.08800	-0.70300
C	-2.69900	0.18500	-0.70400
C	-1.99700	1.40500	-0.10800
C	-0.56500	1.32200	0.04500
C	0.15000	0.17100	-0.21400
H	-0.03600	-1.98000	-0.29400
H	-3.74300	0.19800	-0.37800
O	-2.66700	2.43100	0.18500
O	0.18800	2.38600	0.58100
N	1.50200	0.17600	-0.02300
O	-2.66400	-2.17500	-1.14300
C	-2.23900	-1.50000	1.13300
O	-1.72900	-2.86600	1.27800
C	0.00200	3.71600	-0.01500
H	-1.94000	-3.23000	2.15800
H	-0.45400	-1.15100	-1.79800
H	-2.70500	0.26800	-1.79900
H	1.90700	1.04200	0.31700
H	-2.45900	-3.03600	-0.71400
H	-1.70600	-0.81000	1.79500
H	-3.31200	-1.46500	1.35600
H	0.70900	4.36000	0.50900
H	0.23800	3.69500	-1.08600
H	-1.02400	4.05100	0.13800
C	2.39600	-0.94000	-0.23900
C	3.81800	-0.48500	-0.01800
O	4.16600	0.64800	0.32000
O	4.70000	-1.51200	-0.23800
H	5.62500	-1.21700	-0.09000
H	2.32500	-1.34600	-1.25700
H	2.20600	-1.78100	0.44500

MyG-B (MyG-B)

C	-1.92400	-1.16500	-0.33500
C	-0.41600	-1.18900	-0.61900
C	-2.53200	0.16900	-0.79900
C	-1.81900	1.38800	-0.22000
C	-0.39600	1.26100	-0.00600
C	0.28400	0.07500	-0.18200
H	0.02600	-2.07200	-0.14300
H	-3.58700	0.22700	-0.51900
O	-2.46000	2.44800	0.01000
O	0.38000	2.32300	0.49900
N	1.63100	0.04500	0.04300
O	-2.47000	-2.27400	-1.10700
C	-2.23800	-1.44000	1.14500
O	-3.67400	-1.70500	1.18600
C	0.25500	3.63000	-0.16000
H	-4.01600	-1.70600	2.09800
H	-0.30000	-1.33300	-1.70300
H	-2.47500	0.20200	-1.89500
H	2.05600	0.91700	0.34300
H	-3.36700	-2.46000	-0.75100
H	-1.68200	-2.32600	1.48100
H	-1.97100	-0.58300	1.77300
H	0.51500	3.55200	-1.22300
H	-0.76200	4.00600	-0.04900
H	0.97100	4.27300	0.35200
C	2.49700	-1.10300	-0.10600
C	3.92900	-0.67400	0.10500
O	4.30300	0.46600	0.38600
O	4.78500	-1.73400	-0.05000
H	5.71600	-1.45700	0.09200
H	2.42400	-1.56100	-1.10300
H	2.28100	-1.90300	0.61900

MyG-C (MyG-C)

C	2.02300	1.28050	-1.54960
C	0.67900	0.98560	-2.22350
C	2.71650	-0.03150	-1.20110
C	1.83680	-0.91820	-0.35390
C	0.34400	-0.86400	-0.56460
C	-0.18290	-0.00090	-1.46300
H	0.14950	1.93700	-2.36020
H	3.65440	0.15760	-0.66580
O	2.34340	-1.69880	0.45210
O	-0.51430	-1.73770	0.09900
N	-1.54930	0.01830	-1.71280
O	2.85460	1.97690	-2.49700
C	1.85440	2.20650	-0.32480
O	3.15020	2.55680	0.16830
C	-0.24870	-1.88880	1.48970
H	3.01360	3.12370	0.94890
H	0.84510	0.59580	-3.23720
H	2.98510	-0.58480	-2.10960
H	-2.09460	-0.67870	-1.19320
H	3.59090	2.34840	-1.96760
H	1.36040	3.14000	-0.61510
H	1.28610	1.73870	0.48430
H	0.08280	-0.95210	1.95140
H	-1.18240	-2.18880	1.97520
H	0.47820	-2.68850	1.65580
C	-2.29000	0.78070	-2.71200
C	-3.76270	0.39200	-2.70930
O	-4.30620	-0.42240	-1.97820
O	-4.48070	1.06170	-3.63560
H	-5.39100	0.71310	-3.52530
H	-1.88500	0.57700	-3.70830
H	-2.22260	1.84950	-2.48680

MyG-D (MyG-D)

C	2.30250	1.06500	-1.34240
C	0.95000	0.96770	-2.06700
C	2.81550	-0.33200	-1.03130
C	1.80940	-1.10750	-0.22050
C	0.35000	-0.94030	-0.55010
C	-0.04200	0.02490	-1.41240
H	0.52420	1.97480	-2.15310
H	3.75870	-0.27310	-0.47470
O	2.18520	-1.87480	0.66520
O	-0.62020	-1.73710	0.05270
N	-1.38320	0.18830	-1.73090
O	3.23950	1.68990	-2.23990
C	2.23070	1.94170	-0.07230
O	1.94060	3.28930	-0.45790
C	-0.30500	-3.12650	0.06080
H	1.89360	3.81080	0.36410
H	1.10960	0.63270	-3.10140
H	3.03040	-0.89280	-1.94920
H	-2.03150	-0.45110	-1.25930
H	3.03720	2.64790	-2.19310
H	1.46290	1.60420	0.63000
H	3.20030	1.95840	0.43690
H	-1.24690	-3.68010	0.12430
H	0.20680	-3.43940	-0.85600
H	0.28540	-3.38140	0.94500
C	-2.00700	1.13380	-2.65080
C	-3.51010	0.90420	-2.77240
O	-4.27300	1.53360	-3.49020
O	-3.98590	-0.09210	-1.99010
H	-4.94710	-0.10490	-2.18170
H	-1.57140	1.01910	-3.64810
H	-1.85760	2.15460	-2.28610

MyG-E (MyG-E)

C	2.04400	1.22380	-0.57470
C	0.53390	1.35100	-0.83230
C	2.33700	0.21380	0.52130
C	1.65040	-1.10770	0.27790
C	0.35530	-1.12640	-0.48510
C	-0.18730	0.02160	-0.94640
H	0.05880	1.92320	-0.02510
H	2.00660	0.58130	1.49980
O	2.10720	-2.13580	0.78250
O	-0.35790	-2.31500	-0.62860
N	-1.43730	0.02080	-1.55600
O	2.69310	0.79150	-1.78450
C	2.65460	2.59190	-0.20920
O	2.45750	3.50050	-1.29530
C	0.41380	-3.34240	-1.24570
H	2.90640	4.33090	-1.05320
H	0.38970	1.90980	-1.76530
H	3.41520	0.01620	0.56530
H	-1.89380	-0.89650	-1.60310
H	2.66820	1.56400	-2.38570
H	2.19410	3.01310	0.69030
H	3.73520	2.50210	-0.05380
H	-0.28270	-4.04670	-1.71090
H	1.07070	-2.95100	-2.03070
H	0.98980	-3.89500	-0.49860
C	-2.19050	1.13660	-2.11300
C	-3.55230	0.67740	-2.61700
O	-3.99330	-0.46210	-2.61430
O	-4.29530	1.69220	-3.10860
H	-5.12660	1.25530	-3.39210
H	-1.64560	1.56630	-2.95930
H	-2.35080	1.89640	-1.34150

Table S6: *Optimized Cartesian coordinates of the CI_1 and CI_2, determined at the SA-CASSCF(6,6)/6-31G* theoretical level.*

CI1

-890.875266300052

C	-1.23221878	-1.66828380	-0.12212936
C	-0.50791305	-0.94094530	-1.33708839
C	-2.36423321	-0.77419228	0.43343516
C	-2.20900560	0.75967086	0.41171685
C	-1.22523180	1.26914938	-0.37767376
C	-0.11318674	0.34977513	-0.73865180
H	0.29469820	-1.56036644	-1.73518560
H	-2.55394862	-1.06259279	1.46627476
O	-3.16790404	1.29753430	1.00665981
O	-1.00155277	2.58005784	-0.71756201
N	1.02512157	0.52940693	-0.17500847
O	-1.77503290	-2.85472654	-0.64424781
C	-0.23700149	-2.07273815	0.96986781
O	0.74847471	-2.91296652	0.36917270
C	-2.20881110	3.31965639	-0.65254370
H	1.18706868	-3.40701884	1.05550731
H	-1.26491635	-0.80031944	-2.10013037
H	-3.26265658	-1.02147246	-0.12509948
H	1.13141207	1.46641435	0.20939151
H	-1.06503097	-3.48536363	-0.74765784
H	0.22230682	-1.20522833	1.43651407
H	-0.79805046	-2.61604317	1.72617532
H	-1.94561991	4.34986462	-0.89558397
H	-2.94959203	2.95603558	-1.36025063
H	-2.63796388	3.19867250	0.34193148
C	2.30344708	-0.09324628	-0.51195156
C	3.41222799	0.55833138	0.27967306
O	3.28466349	1.56892799	0.90863013
O	4.55209916	-0.12808450	0.16366785
H	5.22709661	0.34981625	0.64791049
H	2.52637125	0.03287111	-1.56923046
H	2.27531264	-1.15666614	-0.30132311

CI2

		-890.899568789387	
C	-1.27237186	-1.79202439	-0.20631160
C	-0.47334486	-1.16977071	-1.37166306
C	-2.60512760	-1.05252352	-0.04224323
C	-2.41985180	0.45545114	-0.19287237
C	-1.43768360	1.07093255	-0.76391799
C	-0.17665590	0.29561048	-1.16950530
H	0.42644842	-1.75217760	-1.52678463
H	-3.07152410	-1.31429425	0.90467604
O	-3.64638348	0.80577094	0.27679267
O	-1.46842854	2.42037594	-1.07339801
N	0.89073425	0.61020072	-0.29018463
O	-1.64826819	-3.10725526	-0.53453549
C	-0.50695169	-1.83835202	1.11516866
O	0.61445880	-2.66909066	0.90799666
C	-1.44750004	3.28788644	0.02442713
H	1.07526651	-2.79815596	1.72481278
H	-1.08435620	-1.28423600	-2.26153204
H	-3.27162576	-1.37293870	-0.83018710
H	0.96789110	1.60025391	-0.18459428
H	-0.88357336	-3.66135640	-0.43339946
H	-0.20617564	-0.84940773	1.42681154
H	-1.15656224	-2.27727099	1.86782374
H	-1.37591604	4.29438331	-0.36489761
H	-2.35650412	3.21499350	0.62001907
H	-0.60404703	3.09380417	0.67878683
C	2.18882929	0.05202032	-0.58780319
C	3.26626413	0.79050137	0.16480774
O	3.13097181	1.83183080	0.73769899
O	4.44140021	0.15182238	0.09827056
H	5.09749169	0.67364489	0.54772106
H	2.44794629	0.10430914	-1.64588620
H	2.23557956	-0.98900781	-0.30088730

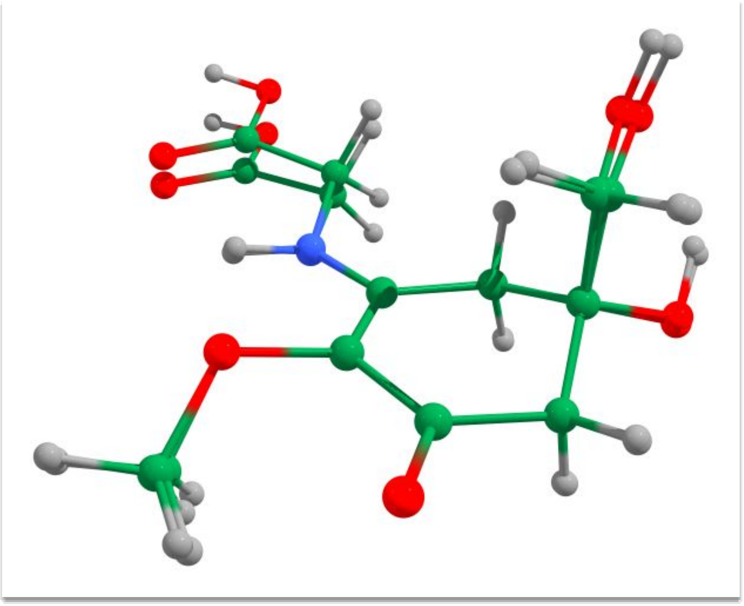

Figure S1: *Comparison the optimized structures of MyG-A at the DFT/B3LYP and MP2 models using cc-pVDZ basis set.*

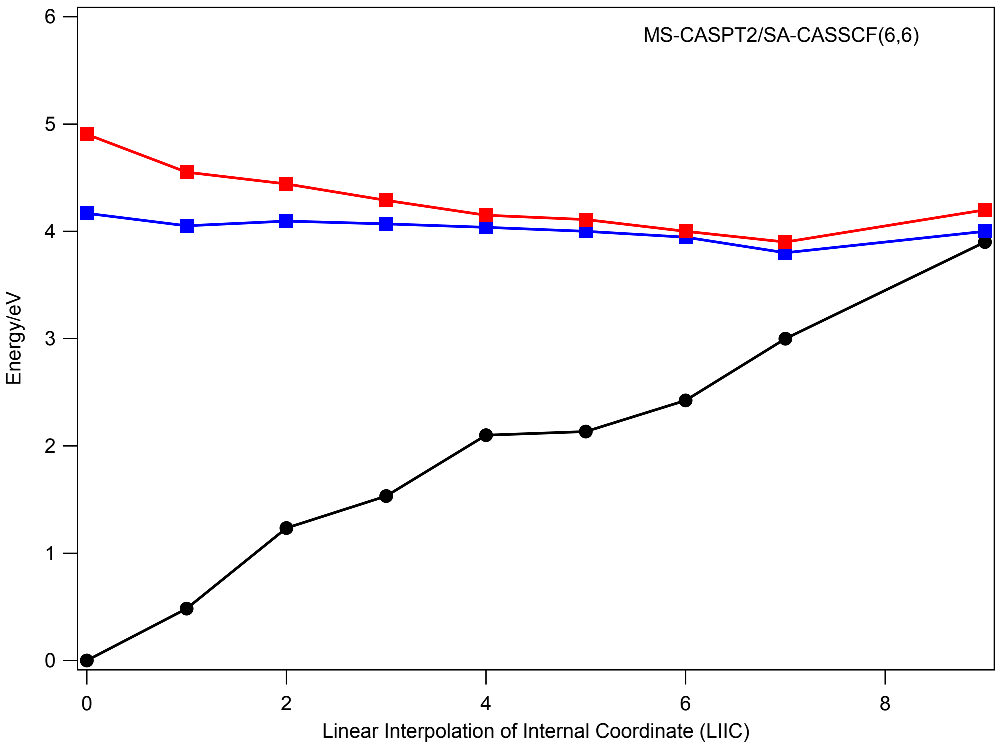

Figure S2:*. Potential energy (PE) profile of the ground (black) and 2 singlet excited states of MyG-A calculated at the MS-CASPT2/SA-CASSCF(6,6)/cc-pVDZ level of theory along the LIIC reaction path.*

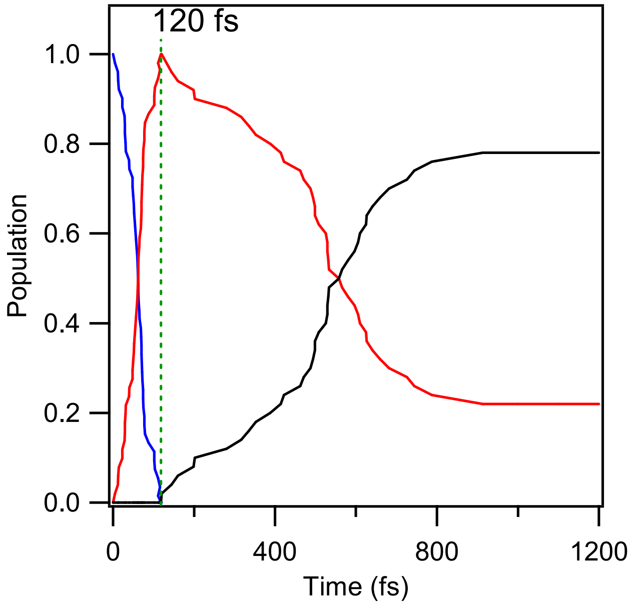

Figure S3: *Time evolution of the nonadiabatic population of the ground state and first excited state from nonadiabatic dynamics simulation for MyG-A.*

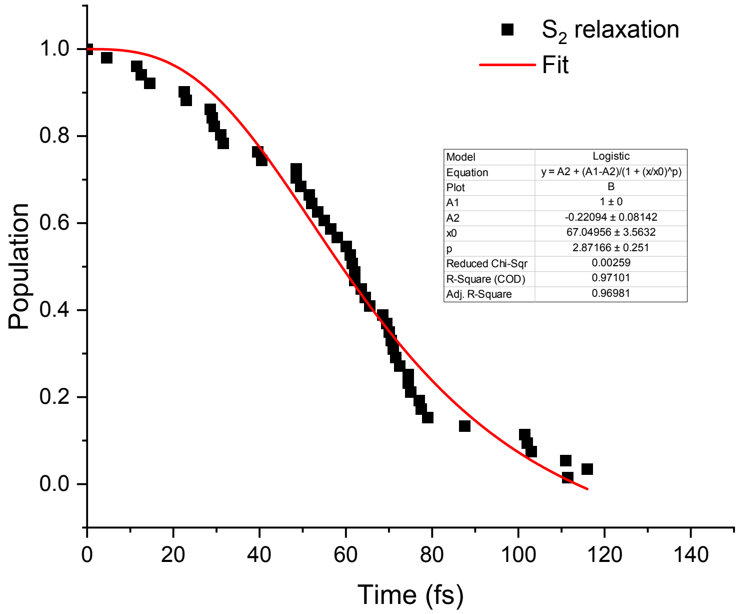

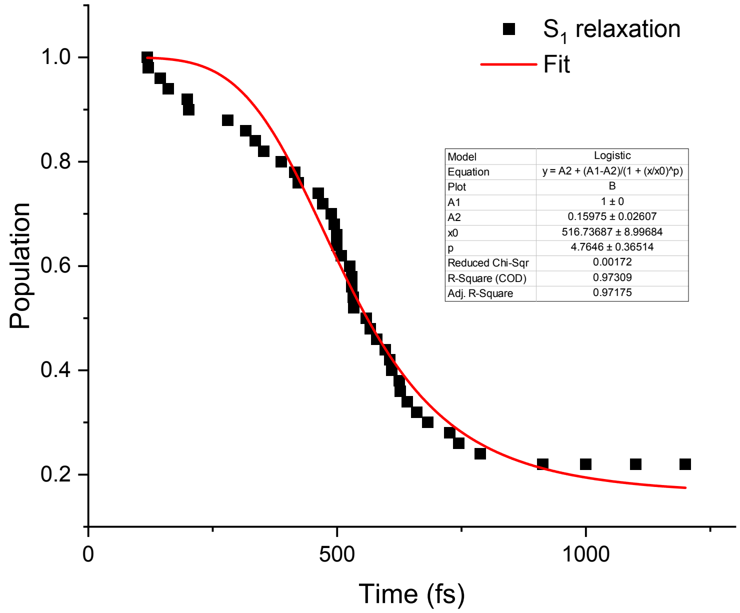

Figure S4: *Approximate determination of the S_2 (top) and S_1 (bottom) excited state lifetime using the logistic fitting algorithm in OriginPro on population versus time.*
